# Assessing Cancer Presence in Prostate MRI Using Multi-Encoder Cross-Attention Networks

**DOI:** 10.3390/jimaging11040098

**Published:** 2025-03-26

**Authors:** Avtantil Dimitriadis, Grigorios Kalliatakis, Richard Osuala, Dimitri Kessler, Simone Mazzetti, Daniele Regge, Oliver Diaz, Karim Lekadir, Dimitrios Fotiadis, Manolis Tsiknakis, Nikolaos Papanikolaou, ProCAncer-I Consortium, Kostas Marias

**Affiliations:** 1 Institute of Computer Science, Foundation for Research and Technology Hellas (FORTH), N. Plastira 100, Vassilika Vouton, 70013 Heraklion, Greece; dimitriadi@ics.forth.gr (A.D.); tsiknaki@ics.forth.gr (M.T.); kmarias@ics.forth.gr (K.M.); 2 Department of Mathematics and Computer Science, Universitat de Barcelona, Gran Via de les Corts Catalanes, 585, L’Eixample, 08007 Barcelona, Spain; richard.osuala@ub.edu (R.O.); dimitri.kessler@ub.edu (D.K.); oliver.diaz@ub.edu (O.D.); karim.lekadir@ub.edu (K.L.); 3 Department of Electrical and Computer Engineering, Hellenic Mediterranean University (HMU), Estavromenos, 71410 Heraklion, Greece; 4 Department of Radiology, Candiolo Cancer Institute–FPO, IRCCS, 10060 Candiolo Torino, Italy; simone.mazzetti@ircc.it (S.M.); daniele.regge@ircc.it (D.R.); 5 Institució Catalana de Recerca i Estudis Avançats (ICREA), Passeig Lluís Companys 23, 08010 Barcelona, Spain; 6 Department of Biomedical Research Institute–FORTH, University Campus of Ioannina, 45110 Ioannina, Greece; fotiadis@uoi.gr; 7 Champalimaud Research, Champalimaud Foundation, 1400-038 Lisbon, Portugal; nickolas.papanikolaou@research.fchampalimaud.org

**Keywords:** prostate cancer, cross-attention, deep learning

## Abstract

Prostate cancer (PCa) is currently the second most prevalent cancer among men. Accurate diagnosis of PCa can provide effective treatment for patients and reduce mortality. Previous works have merely focused on either lesion detection or lesion classification of PCa from magnetic resonance imaging (MRI). In this work we focus on a critical, yet underexplored task of the PCa clinical workflow: distinguishing cases with cancer presence (pathologically confirmed PCa patients) from conditions with no suspicious PCa findings (no cancer presence). To this end, we conduct large-scale experiments for this task for the first time by adopting and processing the multi-centric ProstateNET Imaging Archive which contains more than 6 million image representations of PCa from more than 11,000 PCa cases, representing the largest collection of PCa MR images. Bi-parametric MR (bpMRI) images of 4504 patients alongside their clinical variables are used for training, while the architectures are evaluated on two hold-out test sets of 975 retrospective and 435 prospective patients. Our proposed multi-encoder-cross-attention-fusion architecture achieved a promising area under the receiver operating characteristic curve (AUC) of 0.91. This demonstrates our method’s capability of fusing complex bi-parametric imaging modalities and enhancing model robustness, paving the way towards the clinical adoption of deep learning models for accurately determining the presence of PCa across patient populations.

## 1. Introduction

Prostate cancer (PCa) is a malignancy of the urinary system in men, with the second highest incidence and the fifth leading cause of cancer death among men worldwide in 2020 [1]. The reported incidence rates of PCa are 37.5 per 100,000 in developed countries and 11.3 per 100,000 in developing countries [1], with an estimated worldwide 1.6 million cases and 366,000 deaths annually [2]. Despite these facts, if PCa is detected at the early stages its survival rates increase due to the commonly slow progression of the disease [3]. Therefore, effective monitoring and early diagnosis are crucial for improving patients’ survival.

Currently, accepted clinical strategies for PCa diagnosis include a combination of the prostate-specific antigen (PSA) test, digital rectal exam, trans-rectal ultrasound (TRUS), and pelvic magnetic resonance imaging (MRI). PSA screening, however, results in over-diagnosis, subsequently requiring the patients to undergo invasive prostate biopsy. This procedure causes pain, risk of bleeding, and the potential for over-treatment [4,5]. Pelvic MRI has been increasingly becoming the standard of care for PCa diagnosis in radiology settings. An international standardised guideline to image interpretation (PI-RADS v2) [6] has been developed within the radiology community; however, there remain challenges with inter-observer variability in the use of this guideline [7].

Deep learning (DL) methods, in particular, Convolutional Neural Networks (CNNs), have recently shown promising results in a variety of computer vision tasks, such as segmentation, classification, and object detection [8,9,10]. These methods consist of convolutional layers that are able to extract features from low-level local features to high-level global features from input images in a data-driven way. Therefore, the medical imaging research community has shifted their interest toward DL-based methods for designing computer-aided cancer detection and diagnosis (CAD) tools. CAD algorithms can be associated with two main tasks in PCa: (a) lesion classification, i.e., Computer-Aided Diagnosis (CADx), pathological characterisation of an already known lesion; (b) lesion detection, i.e., Computer-Aided Detection (CADe). The first group includes algorithms that classify manually annotated regions, such as lesion segmentations. The second group includes algorithms that detect and localise PCa lesions and provide the user with probability maps, segmentations, and/or bounding boxes as output.

In their review, Twilt et al. [11] pointed out that from 59 studies, 66% of the articles described lesion classification algorithms while the remaining 34% described lesion detection algorithms. Out of the included studies, 17% described a two-class lesion classification task using a DL approach, with most focusing solely on the algorithm’s stand-alone performance rather than investigating the influence of CADx in a prospective clinical workflow. Among the included DL studies, cohort sizes ranged from 18 to 499 patients (median = 278). Only a limited number of studies involved multi-centric data (16%), whereas the remaining studies utilised retrospectively collected data from a single center (84%).

Patient-level classification frameworks aim to classify patients as having or not having PCa [12]. However, integrating region-of-interest (ROI)-based or slice-level results into patient-level outcomes remains a significant challenge as they usually rely on basic merging methods such as simple voting [13]. Yoo et al. [14] proposed a CNN-based approach to detect clinically significant PCa using diffusion-weighted imaging (DWI). Features from CNN outputs were selected via decision trees and used to train a random forest classifier, achieving receiver operating characteristic curves (AUCs) of 0.87 (slice-level) and 0.84 (patient-level). The study used a private dataset with 319 patients for training and 108 for testing.

Aldoj et al. [15] proposed a 3D multi-modal CNN architecture that uses lesion center parameters and different 3D volume combinations, T2-weighted images (T2ws), apparent diffusion coefficient maps (ADCs), and diffusion-weighted images (DWIs), as input channels to classify clinically significant and insignificant PCa lesions. Their best model achieved an AUC of 0.91, sensitivity of 0.81, and specificity of 0.91. The study used the publicly available PROSTATEx dataset [16] with 175 patients used for model training and 25 patients for testing.

In this paper, we propose a novel multi-encoder-cross-attention 3D convolutional architecture to predict patient-level presence or absence of PCa by classifying whole MRI volumes. Our approach addresses the issue of isolated standalone solutions per modality by integrating multiple modalities into a single model to learn multi-faceted patterns from combined patient data thereby achieving higher efficiency while improving classification performance. Additionally, and in order to promote further collaboration, comparison, and development of task-specific model architectures, we benchmark the performance of our proposed architectures on 975 unseen retrospective cases and 435 unseen prospective cases. To the best of our knowledge, this is the first type of work, at such large scale, seeking to address a pressing real-world clinical scenario: how to stratify men with prostate hypertrophy or inflammation despite the high PSA values and those who should undergo additional diagnostic tests (e.g., biopsy) to identify if suspicious findings identified on MRI are clinically significant. It is to be noted that this research provides relevant clinical insight on whether prostate cancer is present rather than focusing on the separate task of tumour localisation within the gland. With definition of tumour extension and localisation being out of scope for this article, we advocate per-lesion analysis as a subsequent step in our proposed workflow to address issues such as unilateral or bilateral localisation of tumours and possibly also extracapsular extension, which are factors that affect the T-stage (primary tumour staging (T)). The main contributions of this paper are summarised as follows:

- Establishing a new performance benchmark adopting and processing the largest and most diverse multi-centric PCa MR image database worldwide (6458 retrospective cases and 436 prospective cases)
- Design and implementation of a classification scheme to distinguish pathologically confirmed PCa patients from conditions with no suspicious PCa findings from whole MRI volumes
- Introducing a novel multi-encoder-cross-sequence attention neural network architecture for bpMRI data, enhanced by the integration of clinical variables
- The proposed architecture shows better generalisation performance, AUC of 0.87, in an out-of-distribution setting compared to radiologists (AUC of 0.76) and other architectures frequently used in the literature (AUC of 0.80 and 0.84), respectively.
- Thorough fairness analysis to identify potential biases in the modelling process

## 2. Materials and Methods

### 2.1. Patient-Level PCa Classification Workflow

**Figure 1:**
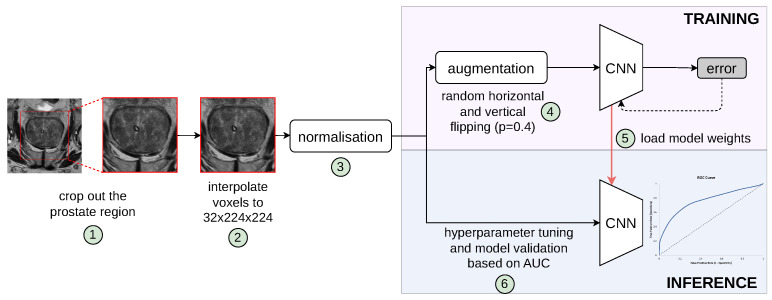
Pipeline of our proposed patient-level classification scheme to distinguish pathologically confirmed PCa patients from conditions with no suspicious PCa findings. Individual steps in the pipeline are indicated by numbers circled in green, which are described in the text. The validation set was used for hyperparameter tuning and model selection; thus, the best model, determined by the lowest validation loss, was evaluated on the unseen retrospective and prospective test cohorts.

We define the patient-level differentiation of suspicious findings from no PCa findings as a binary classification task. Specifically, while the input of the proposed model is a whole 3D MRI volume comprising the prostate gland and surrounding tissues, the output is a discrete label of value either 0 for PCa negative or 1 for PCa positive. Figure 1 shows the complete pipeline of our proposed patient-level classification scheme. It should be noted that the formulation of our classification task is fundamentally different from common tasks found in the literature, such as lesion classification, lesion detection [11], clinically significant prostate cancer detection [17], prediction of PCa aggressiveness [18], and classification of a segmented ROI [19]. In order to train learning algorithms for these two groups, manually annotated regions of interest (lesions) are required, while cases without PCa findings are ignored due to being outside the scope of the required training data characteristics. Bridging this gap, our networks are optimised in an end-to-end training framework provided with the information of a patient’s presence/absence of PCa based on a biopsy-proven ground truth label.

### 2.2. Clinical Variables

PSA is a widely used biomarker for PCa detection; however, it can be influenced by other factors, such as benign prostatic hyperplasia (BPH) or prostatitis. A high PSAD can suggest cancer more strongly than a high PSA alone, especially in men with larger prostates [20]. Combining both can potentially improve the model’s ability to distinguish between cancerous and benign conditions. Additionally, higher PSAD has been associated with more aggressive prostate cancers [21].

To accurately calculate PSA density, the prostate volume must be precisely determined. First, we count the total number of voxels representing the prostate tissue, denoted by $N_{\mathrm{voxels}}$. The volume of a single voxel, $V_{\mathrm{voxel}}$, is computed from the physical dimensions of each voxel $\left(d_{x},d_{y},d_{z}\right)$ provided by the T2w DICOM.

Finally, the prostate volume is used in the denominator with the patient’s PSA level to calculate the PSA density. To this end, a segmentation of the whole prostate gland was performed on the ProstateNet dataset using the T2w sequence, utilising the nnU-Net V2 model [22] available through the open-source MONAI framework [23]. The random splits for training, validation, and test sets were determined by the nnU-Net V2 framework, comprising 391, 98, and 55 entries, respectively. The performance evaluation, based on a 5-fold cross-validation on the ProstateNet dataset [24], yielded a mean Dice Similarity Coefficient (DSC) of 0.9257, with a standard deviation of ±0.0013. According to a recent systematic review for a whole prostate gland segmentation [25], most deep learning algorithms (39 of 42 [93%]) had a DSC at or above expert level (DSC ≥0.86). Thus, our statistics indicate a high level of accuracy in the segmentation of prostate images, demonstrating the effectiveness of the model employed in capturing the relevant anatomical structures. This precise segmentation enables us to accurately estimate PSA density across the entire dataset. In addition to PSA density, clinical variables, such as age and PSA, are considered in the study. All variables are normalised to the range [−1, 1] to ensure uniformity and comparability in the model’s input data. This normalisation is essential for the subsequent integration of these variables with the feature vectors derived from imaging data.

### 2.3. Deep Convolutional Networks

All 3D models implemented in this study leverage a VGG backbone architecture [26], while each bi-parametric volume is resampled into 3.0, 0.5, 0.5 mm spacing, and a central crop of 32 × 224 × 224 matrix covering the prostate is extracted, as shown in steps 1 and 2 in Figure 1. Importantly, all sequences in the dataset are individually normalised to values between 0 and 1 using a patient-level min-max normalisation strategy. This normalisation is executed by subtracting the minimum pixel intensity value from each voxel and dividing by the range of the pixel intensities within each individual image.

#### 2.3.1. 3-Channel Architecture

**Figure 2:**
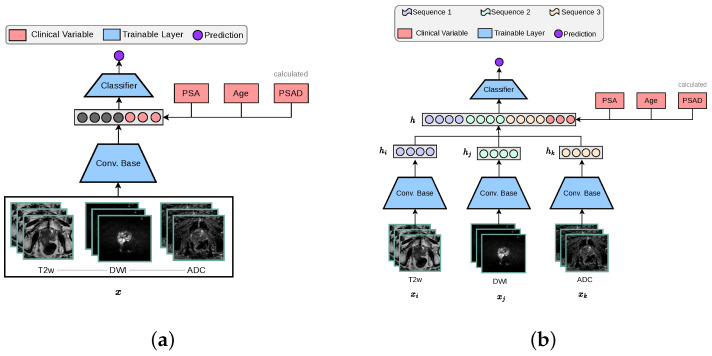
The two baseline 3D architectures implemented for this study, in accordance with other works in the literature [14]. The ‘3-Channel’ network modifies a 3D CNN architecture to process MRI volumes as channel-wise concatenated inputs. For our ‘Multi-Encoder-Fusion’ networks, we propose an early fusion (feature-level fusion) strategy of the latent representations retrieved from the 3D CNN encoders. (**a**) 3-Channel network. (**b**) Multi-Encoder (ME) Fusion network.

The standard 3D CNN VGG architecture was modified to process MRI volumes by stacking T2w, DWI-high-b-value, and ADC sequences into the three input channels. These channels were originally configured for RGB colour data in traditional image processing applications, as depicted in Figure 2a. The encoder is designed with multiple stages, each containing an increasing number of feature maps ([64, 128, 256, 512]) and blocks per stage ([1, 2, 3, 4]). Each stage involves convolutions, batch normalisation (BN), ReLU activations, and includes a dropout rate of 0.5, followed by adaptive pooling (GlobalMaxAvgPool) that combines features through max and average pooling to produce a 512-dimensional feature vector. This vector concatenated with clinical variables is subsequently processed by a linear layer to generate a one-dimensional prediction.

#### 2.3.2. Multi-Encoder-Fusion Architecture

The multi-encoder-fusion architecture leverages an early fusion strategy. Early fusion refers to combining different modalities at the input level and raw data can be fused directly without any pre-processing. In this architecture, the three different input sequences ($x_{i}$, $x_{j}$, $x_{k}$) are passed through separate encoders to extract their respective 512-dimensional feature vectors ($h_{i}$, $h_{j}$, $h_{k}$). These vectors are then concatenated along with clinical variables, forming a 1539-dimensional representation of all inputs *h*, which is subsequently fed into a classifier as depicted by Figure 2b. This approach ensures an efficient feature integration while maintaining the model’s capacity to capture MRI sequence-specific nuances.

#### 2.3.3. Multi-Encoder-Cross-Attention-Fusion Architecture

**Figure 3:**
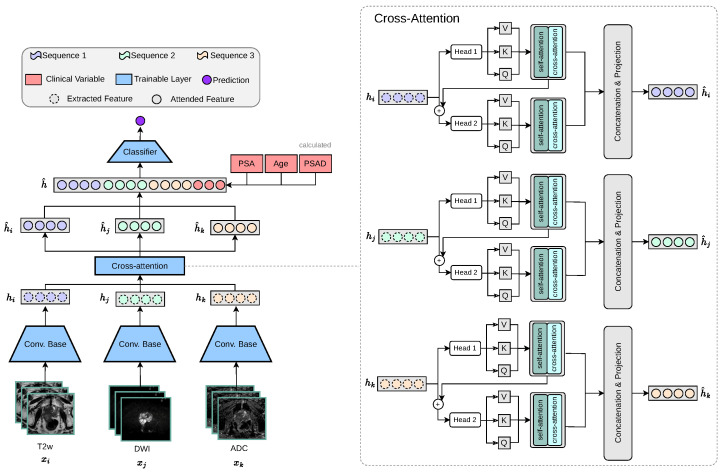
Our proposed multi-encoder-cross-attention-fusion architecture is specifically designed to enrich the feature representations of each MRI sequence by incorporating information from the other MRI sequences. The cross-attention module is depicted as a generic representation, making it applicable to any number of attention heads.

PCa commonly manifests as hypointense areas (lower signal intensity compared to surrounding normal tissue) on T2W, and as hyperintense areas (higher signal intensity compared to surrounding normal tissue) on DWI with high diffusion weighting (b-values). In addition, these lesions typically show low ADC values on the corresponding maps. Thus, these modality-specific visual correlations between MRI sequences serve as the intuition behind our proposed cross-attention fusion mechanism. The core idea of our proposed architecture is to enrich the feature representations of each input sequence ($x_{i}$, $x_{j}$, $x_{k}$) by incorporating information from the other sequences in a meaningful and efficient way. To achieve this, we introduce a novel cross-sequence attention mechanism specifically designed for the task at hand, as illustrated in Figure 3.

**Figure 4:**
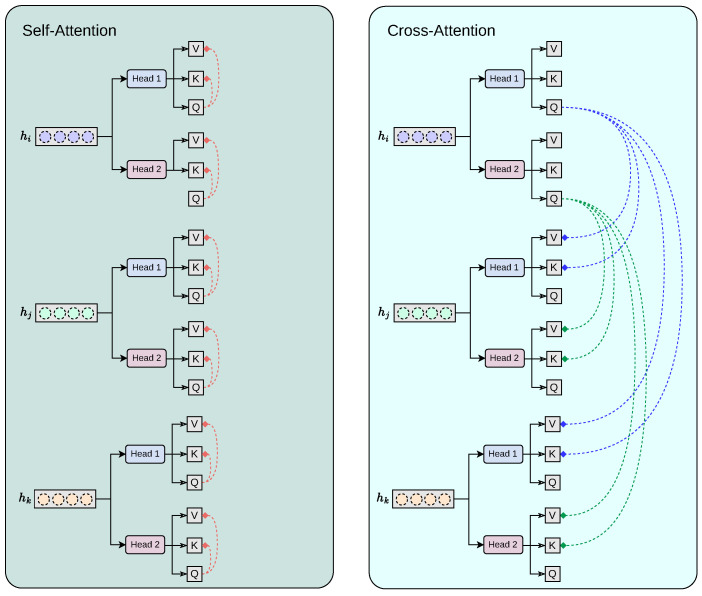
Our proposed cross-modality fusion mechanism combines two separate modules: self-attention and cross-attention to learn accurate latent representations per-feature (self-attention), per-modality (head k to head k + 1 feature concatenation), and cross-modality (cross-attention) representation.

Our proposed cross-modality fusion mechanism combines two separate modules: self-attention and cross-attention to learn accurate latent representations per-feature (self-attention), per-modality (head k to head k + 1 feature concatenation), and cross-modality (cross-attention) representation, as seen in Figure 4. It takes as input a set of extracted feature vectors $\left\{h_{i},h_{j},h_{k}\right\}$, each of dimension $R^{d}$. The output consists of three “attended” vectors $\left\{{\hat{h}}_{i},{\hat{h}}_{j},{\hat{h}}_{k}\right\}$, still in $R^{d}$. Each feature vector, extracted from the convolutional base $h_{i}$, acts as the query for one of the attention heads, while the keys and values are derived from all feature vectors $\left\{h_{i},h_{j},h_{k}\right\}$. Additionally, the mechanism includes a cascading step where the result of each attention head is added back into the input feature for the next head, allowing iterative refinement across heads. For each feature vector $h_{i}\in R^{d}$ corresponding to sequence i∈{1,2,3}, we first apply a linear transformation to obtain the query vector:

$$Q_{i}=h_{i}W_{i}^{Q},$$

where $W_{i}^{Q}\in R^{d\times d}$ is a learnable parameter, keeping $Q_{i}\in R^{d}$. Similarly, for each j∈{1,2,3}, we compute the keys and values:

$$K_{j}=h_{j}W_{j}^{K},\;V_{j}=h_{j}W_{j}^{V},$$

with $W_{j}^{K},W_{j}^{V}\in R^{d\times d}$ being learnable parameters. Thus, at each step *i*, the query $Q_{i}$ is derived from $h_{i}$ alone, while the keys and values are the collection of $\left\{K_{1},K_{2},K_{3}\right\}$ and $\left\{V_{1},V_{2},V_{3}\right\}$, respectively.

Our module employs H=2 attention heads. We split the transformed queries, keys, and values into *H* sub-vectors each of dimension $d_{h}=\frac{d}{H}$. For each head h∈{1,…,H}, the transformations are as follows:

$$Q_{i}\in R^{d}\;⟶\;\left[Q_{i}^{1},Q_{i}^{2},\cdots ,Q_{i}^{H}\right],$$

$$K_{j}\in R^{d}\;⟶\;\left[K_{j}^{1},K_{j}^{2},\cdots ,K_{j}^{H}\right],$$

$$V_{j}\in R^{d}\;⟶\;\left[V_{j}^{1},V_{j}^{2},\cdots ,V_{j}^{H}\right].$$

For a given head *h* when sequence *i* serves as the query source, the attention scores are computed by comparing $Q_{i}^{h}$ to all keys $\left\{K_{1}^{h},K_{2}^{h},K_{3}^{h}\right\}$. Specifically, we calculate the unnormalised attention score from query *i* to key *j* as follows:

$$\alpha_{i\to j}^{h}=\frac{\left(Q_{i}^{h}\right)\;\cdot \;{\left(K_{j}^{h}\right)}^{⊤}}{\sqrt{d_{h}}},$$

where $\alpha_{i\to j}^{h}$ represents the attention score. Applying the softmax function over j∈{1,2,3} yields the attention probabilities:

$${\mathrm{AttnProb}}_{i\to j}^{h}=\frac{\exp(\alpha_{i\to j}^{h})}{\sum_{m=1}^{3}\exp\left(\alpha_{i\to m}^{h}\right)}.$$

The output of each head is then a weighted sum of the corresponding values:

$${\mathrm{HeadOutput}}_{i}^{h}=\sum_{j=1}^{3}{\mathrm{AttnProb}}_{i\to j}^{h}\;V_{j}^{h}.$$

After computing the outputs for all heads, we concatenate them along the feature dimension:

$${\mathrm{ConcatOutput}}_{i}=\overset{H}{\underset{h=1}{⨁}}{\mathrm{HeadOutput}}_{i}^{h}\;\in R^{d},$$

where ⊕ denotes concatenation.

### 2.4. Experimental Setting

All networks were trained using the Adam optimiser [27] with a learning rate of $3\times 10^{-5}$ including L1 and L2 regularisation strategies, assigning them a value of $3\times 10^{-5}$. Data augmentation is applied, including random flip and rotation. During training, gradient descent optimises the network weights according to the error calculated by the binary cross-entropy loss function. After each training epoch, the model performance on the validation set was assessed by the AUC.

Model testing was carried out in the previously unseen test cohorts using the best performing model on the validation set. The experiments were conducted on a workstation featuring an Intel^®^Core™ i9-10940X @ 3.30 GHz processor, an NVIDIA GeForce RTX 3090 GPU and with 24 GB of RAM, running a 64-bit operating system. PyTorch 2.0.1 was used during the experiments, written in Python 3.9.

### 2.5. Prostate MRI Dataset

**Figure 5:**
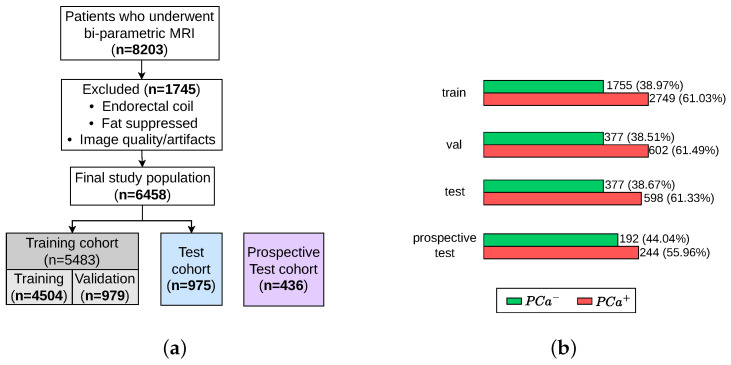
Patient selection process for training, validation, test, and retrospective test sets. (**a**) Flow diagram of the study design. (**b**) Number of patients with and without PCa.

Data are obtained from the ProstateNET Imaging Archive [24]. The platform hosts the largest collection of anonymised PCa multi-parametric (mp) MRI imaging data, in line with European data protection legislation (GDPR). The archive contains imaging data from 13 different clinical centers, 3 scanner manufacturers, and 27 scanner models, making it the largest and most diverse PCa MR image dataset worldwide so far. A total of 6458 retrospective bi-parametric prostate MRI examinations are included in this study. They were split into 70% training set (4504 exams), 15% validation set (979 exams), and 15% test set (975 exams). The ratio between the PCa-positive examinations and PCa-negative examinations were kept roughly similar throughout all subsets, as seen in Figure 5b. Furthermore, 436 bi-parametric prostate MRI examinations from the prospective cohort of ProstateNET are included as an additional hold-out test set (Figure 5a).

## 3. Experimental Results

### 3.1. Evaluation Metrics

The assessment of the models was conducted using two hold-out test sets, as seen in Figure 5a. For each test set, the sensitivity, specificity, positive predicted value (PPV), and negative predicted value (NPV) were calculated based on the recorded true positives (TP), false positives (FP), true negatives (TN), and false negatives (FN) of each class. In addition, from the predicted probabilities, we computed the Area Under the Receiver Operating Characteristic (AUROC). The ROC curve plots the True Positive Rate against the False Positive rate. The AUROC of a random classifier is 0.5, and a perfect classifier has an AUROC of 1. Sensitivity, or the true positive rate, quantifies how well a model identifies true positives. Sensitivity measures the proportion of subjects with an actual positive outcome who are correctly given a positive prediction by the DL model (suspicious findings). Specificity, or the TN rate, quantifies how well a model identifies true negatives. Specificity measures the proportion of subjects with an actual negative outcome who are correctly given a negative prediction by the DL model (non-suspicious PCa findings). Finally, confidence intervals (CI) play an important role in deep learning that help us in finding the uncertainty in model predictions and parameter estimates. A confidence interval (CI) is a range of values that likely contains the true population parameter, such as the mean or proportion based on a sample. A 95% confidence interval of [0.88–0.91] for the MECA-Fusion model accuracy in Table 1 means that if we repeated the process multiple times, about 95% of the intervals would contain the true accuracy. This helps us assess model reliability and make informed decisions.

Although sensitivity and specificity are highly relevant statistical parameters for assessing the performance of a diagnostic test, in real-world practice, it is often more meaningful to predict whether a particular patient will truly have the disease based on a positive or a negative prediction made by the DL model. To this end, PPV and NPV reflect the proportion of positive and negative results that are TP and TN, respectively. In other words, PPV answers the question, ‘if I have a positive DL prediction, what is the probability that I actually have the disease?’. Conversely, NPV answers the question, ‘if I have a negative DL prediction, what is the probability that I actually do not have the disease?’. PPV and NPV depend on the pre-test probability, which is determined by baseline risk factors, such as disease prevalence [28], and can be computed as follows:

(1) $$PPV=\frac{Sensitivity\times Prevalence}{Sensitivity\times Prevalence+(1-Specificity)\times (1-Prevalence)}$$

(2) $$NPV=\frac{Specificity\times (1-Prevalence)}{\left(1-Sensitivity\right)\times Prevalence+Specificity\times (1-Prevalence)}$$

### 3.2. Results

**Figure 6:**
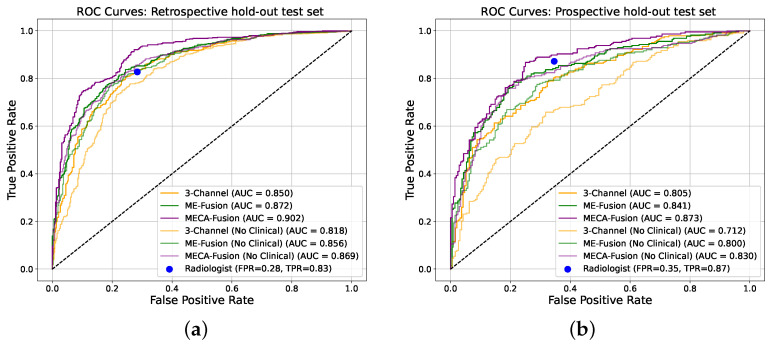
Receiver operating characteristic (ROC) curves of the deep learning models making independent predictions on the two different hold-out test sets. (**a**) Retrospective holdout test set. (**b**) Prospective holdout test set.

The performance of our architecture, equipped with the proposed cross-modality fusion mechanism was thoroughly evaluated against the two baseline architectures (‘3-Channel’ and ‘Multi-Encoder-Fusion’) across two distinct cohorts from ProstateNET, as seen in Figure 6. This evaluation not only assesses the accuracy of the three different models, but also its clinical applicability in distinguishing between cancerous and non-cancerous prostate MRI scans compared to radiologists.

In the retrospective test cohort, our proposed architecture, ‘Multi-Encoder-Cross-Attention-Fusion (MECA-Fusion)’, together with clinical variables, achieved the highest AUC with 0.90 (95% Confidence Interval (CI): 0.88–0.91) at patient level, as seen in Figure 6a. Our proposed architecture also achieves the highest sensitivity among all models with 0.91. Even without the use of clinical variables, our proposed method constantly outperforms the two baseline methods in terms of AUC, sensitivity, PPV, and NPV, as seen in Table 1.

The prospective test cohort presented a different challenge, as seen in Table 2. Our proposed architectures continues to outperform the ‘3-Channel’ and ‘Multi-Encoder-Fusion’ architectures in terms of AUC, sensitivity, PPV, and NPV, as seen in Figure 6b. This illustrates our model’s robustness and applicability to varying disease prevalence. Furthermore, the highest PPV for this cohort indicates the ability of our proposed model to reduce FP effectively.

### 3.3. Statistical Analysis

The results of the DeLong statistical tests [29] across both retrospective and prospective cohorts highlight the superiority of our proposed architecture. Specifically for the retrospective cohort, our model demonstrated significant performance gains over the baseline model with stacked channels in the absence of clinical data (*p* = 0.005) and remained superior when clinical data were incorporated (*p* = 0.001). While our proposed model outperformed the fusion model without clinical data, the difference was not statistically significant (*p* = 0.058). However, when clinical data were added, our proposed model significantly exceeded the performance of the fusion model (*p* = 0.00004). Furthermore, integrating clinical data led to significant improvements for each model variant, including the baseline model with stacked channels (*p* = 0.0009), the baseline multi-encoder-fusion model (*p* = 0.036), and our proposed model (*p* = 0.03), underlining the importance of clinical information.

In the prospective cohort, our proposed model continues to outperform the baseline model with stacked channels both without (*p* = 0.001) and with (*p* = 0.01) clinical data. A notable advantage was also observed when comparing the proposed model to the baseline multi-encoder-fusion model without (*p* = 0.03) and with (*p* = 0.005) clinical data, indicating consistent gains across different fusion strategies. Similar to the retrospective cohort, adding clinical data significantly improved performance for all model variants, including the baseline model with stacked channels (*p* = 0.0036) and baseline multi-encoder-fusion model (*p* = 0.005). Importantly, our proposed model benefited significantly from the inclusion of clinical data (*p* = 0.005), highlighting its capacity to integrate imaging and clinical features more efficiently.

### 3.4. Fairness and Sub-Cohort Analysis

**Figure 7:**
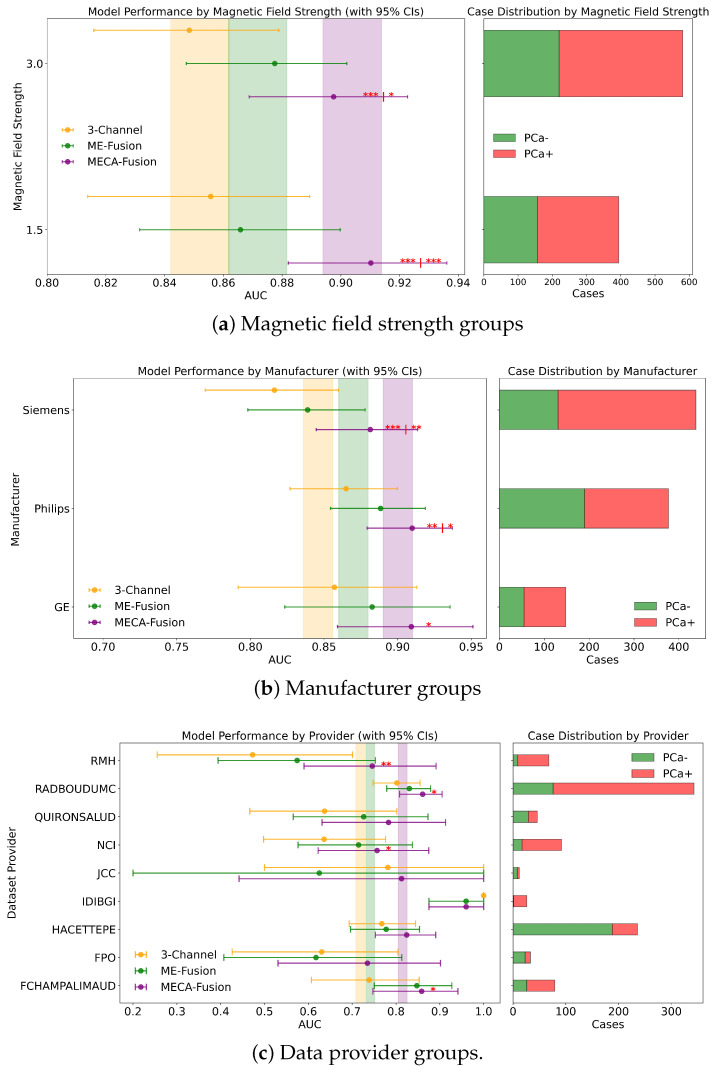
Fairness analysis for sub-cohorts stratified by (**a**) magnetic field strength, (**b**) manufacturer, and (**c**) data provider. Point-and-range plots show model performance (mean and 95% CI), with colored vertical lines indicating expected performance on the retrospective test set. Horizontal bar plots display target and stratum counts. Asterisks (*) denote statistical significance compared to other models, with *** (p≤0.001), ** (p≤0.01), and * (p≤0.05). If no ‘|’ appears, significance is relative to the 3-channel model; if ‘|’ is present, it applies to both compared models.

**Figure 8:**
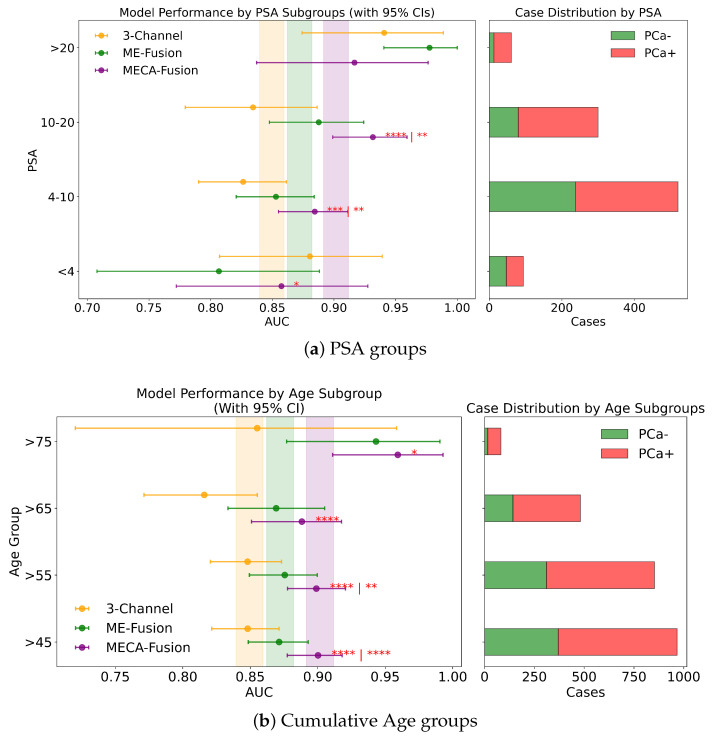
Fairness analysis for sub-cohorts stratified by (**a**) PSA and (**b**) age. Point-and-range plots show model performance (mean and 95% CI) with vertical lines indicating expected performance on the retrospective test set. Horizontal bar plots display target and stratum counts. Asterisks (*) denote statistical significance, with **** (p≤0.0001), *** (p≤0.001), ** (p≤0.01), and * (p≤0.05). If no ‘|’ is shown, significance is relative to the 3-channel model; if ‘|’ appears, it applies to both compared models.

Different DL architectures tend to perform unevenly based on varying forms and levels of stratification. Analyzing this is crucial for identifying potential biases in the modelling process. Additionally by understanding for which sub-cohorts different models perform better, it is possible to better define real world scenarios where they can be applied effectively. In this section, different stratifying variables—PSA, age, dataset provider, magnetic field, and manufacturer—are analysed for all three DL models. The results for the retrospective and prospective hold-out test sets are displayed in Figure 7 and Figure 8 and Appendix A, respectively.

#### 3.4.1. PSA

In this study, we assess the performance of our models across different PSA subgroups, categorising them according to the D’Amico risk criteria. This approach divided the groups into three classes: [0 ng/mL–10 ng/mL), [10 ng/mL–20 ng/mL], and >20 ng/mL. For the retrospective cohort utilising both imaging and clinical data, the MECA-Fusion model demonstrated significant superiority in the [0 ng/mL–10 ng/mL) PSA category compared to the other models, with a *p*-value < 0.001. In the [10–20] category, the MECA-Fusion model also showed significant improvements, with *p*-values < 0.0001 against the 3-Channel model and <0.01 against the ME-Fusion model. However, in the >20 ng/mL PSA category, the MECA-Fusion model did not achieve statistical significance, and the AUCs for the other models were notably higher. When only imaging data were considered, the MECA-Fusion model retained its significance in the same PSA categories except for the [0 ng/mL–10 ng/mL) category, where it did not show statistical significance against the ME-Fusion model.

For the prospective cohort analysis, incorporating imaging and clinical data (Figure A2c) revealed that the MECA-Fusion model was significant in the [0 ng/mL–10 ng/mL) category with a *p*-value < 0.05 and in the [10 ng/mL–20 ng/mL] category only against the 3-Channel model with a *p*-value < 0.05. The model maintained a higher AUC compared to the ME-Fusion model, although this was not statistically significant. In the >20 category, the MECA-Fusion model showed higher AUCs compared to the retrospective cohort, though it did not reach statistical significance. With only imaging data, the MECA-Fusion model in the [0 ng/mL–10 ng/mL) category was significantly better than both the 3-Channel and ME-Fusion models, with *p*-values < 0.0001 and <0.05, respectively. It also showed significance in the [10 ng/mL–20 ng/mL] category against the 3-Channel model with a *p*-value < 0.05. Interestingly, excluding clinical data appeared to reduce the MECA-Fusion model’s performance, especially evident as it yielded a lower AUC compared to the ME-Fusion model.

Further subdividing the PSA criteria into a more granular four-class system that includes a lower risk category of <4 ng/mL, the retrospective analysis with both imaging and clinical data showed (Figure 8a) that the MECA-Fusion model was significantly better in the lowest risk category (<4) with a *p*-value < 0.05 compared to the ME-Fusion model. In the [4 ng/mL–10 ng/mL) category, the MECA-Fusion model continued to demonstrate statistical significance, with *p*-values < 0.001 against the 3-Channel and <0.01 against the ME-Fusion model. The removal of clinical data had a detrimental effect, rendering the MECA-Fusion model non-significant in the lowest risk category and only significant against the 3-Channel model in the [4 ng/mL–10 ng/mL) category with a *p*-value < 0.01. Prospectively, with the inclusion of both imaging and clinical data (Figure A3a), the MECA model was only significant in the [4 ng/mL–10 ng/mL) category with a *p*-value < 0.01. When clinical data were removed, the model performed significantly better in the lower risk category with a *p*-value < 0.05 against the 3-Channel model and retained better AUC and significance in the [4 ng/mL–10 ng/mL) category with a *p*-value < 0.001.

This comprehensive analysis underscores that including clinical data may not always enhance the performance of the MECA model across all PSA subgroups, particularly in certain lower risk categories. This indicates a complex relationship between clinical data and how well the model works, emphasising the importance of customising approaches based on PSA levels and the specific data at hand.

#### 3.4.2. Age

Performance of the models was assessed for different groups of patients stratified by age according to the following criteria:

- Patients who are less likely to undergo surgery (age group > 75), due to their health status and life expectancy, compared to men with ⩽75 years
- Men candidates for prostate cancer screening programs, according to urology guidelines, i.e., aged 55–65 years, compared to the other age groups, i.e., <55 or >65 years
- Increasing age groups (i.e., >45 vs. >55 vs. >65 vs. >75) to identify possible trends of the models performance on specific dichotomised patients groups
- Increasing age groups, classified as young (45–55), middle age (55–65), and older (65–75) patients

Across all four stratified groups (retrospective vs. prospective, with and without clinical data), the proposed MECA-Fusion model consistently achieves the highest AUC in both subcohorts.

**Patients who are less likely to undergo surgery**. In the retrospective setting that combines imaging and clinical data, MECA-Fusion shows statistically significant improvements over both the baseline 3-Channel and ME-Fusion models with *p* < 0.0001. A similar pattern emerges in the retrospective imaging-only plot, where MECA-Fusion again outperforms the other approaches—particularly in the ≤75 subgroup, with *p* < 0.05 or *p* < 0.001. Moving to the prospective setting, the MECA-Fusion model continues to dominate in the imaging + clinical data plot, evident by the significance markers when comparing it against 3-Channel and ME-Fusion (*p* < 0.01). Even in the prospective imaging-only scenario (Figure A3c), where overall margins are tighter, MECA-Fusion frequently retains a significant edge. These plots underline that the MECA-Fusion architecture not only benefits from integrating clinical data (where it tends to show greater gains) but also exhibits robust performance advantages in imaging-based workflows, specifically for patient who might undergo surgery.

**Screening Candidate Groups (<55 years; 55–65 years; >65 years)**. Across all three age subgroups (<55, 55–65, and >65), the MECA-Fusion architecture demonstrates the highest AUC in both retrospective and prospective settings, whether clinical features are included or not. In the retrospective imaging-plus-clinical-data plots, one observes the significance of the cross-attention mechanism, underscoring its superiority over both models in key subgroups—particularly in the middle (55–65) range, but also for those <55 and >65 (*p* < 0.05; *p* < 0.01; *p* < 0.01; *p* <0.0001). Similar patterns emerge in the retrospective imaging-only scenario, although in the <55 cohort the performance is significantly dropped. Meanwhile, in the prospective analysis (Figure A3b), the MECA-Fusion model again achieves consistently higher AUCs and maintains significance against 3-Channel and ME-Fusion in multiple subgroups, reinforcing its robustness when faced with out-of-distribution data. Particularly low *p*-values (*p* ≤ 0.0001) indicate simultaneous significance comparisons (first vs. 3-Channel, second vs. ME-Fusion). Taken together, these plots illustrate that MECA-Fusion confers meaningful performance gains across a range of ages, when clinical variables are incorporated.

**Cumulative Age Groups (>75 years vs. >65 years vs. >55 years vs. >45 years)**. In our analysis, we stratified patient age into four categories: >75, >65, >55, and >45 years, to assess our models across these diverse age subgroups. In the retrospective cohort incorporating imaging and clinical data, the MECA model demonstrated statistical superiority against the 3-Channel model across all age groups, and notably outperformed the ME-Fusion model in the >55 and >45 categories, as seen in Figure 8b. This highlights MECA-Fusion’s robust performance, particularly as younger age subgroups were included. When examining imaging data alone, MECA-Fusion maintained its advantage in the >75 category but lost statistical significance and showed a lower AUC compared to the ME-Fusion model. This suggests that the omission of clinical data notably decreases its effectiveness, especially in older age groups. In the prospective dataset, with both imaging and clinical data included, MECA-Fusion excelled in the younger age groups (>45 and >55), demonstrating significant improvements over both baseline models with *p*-values < 0.01. However, in the >65 group, it only surpassed the 3-Channel model, and in the >75 category, it did not achieve statistical significance. Focusing on imaging data alone in the prospective analysis, MECA-Fusion continued to outperform the 3-Channel model across nearly all age groups (Figure A2a). It did, however, lose its edge against the ME-Fusion model in the >55 group, suggesting that the exclusion of clinical data specifically undermines performance in this age cohort.

**Figure A1:**
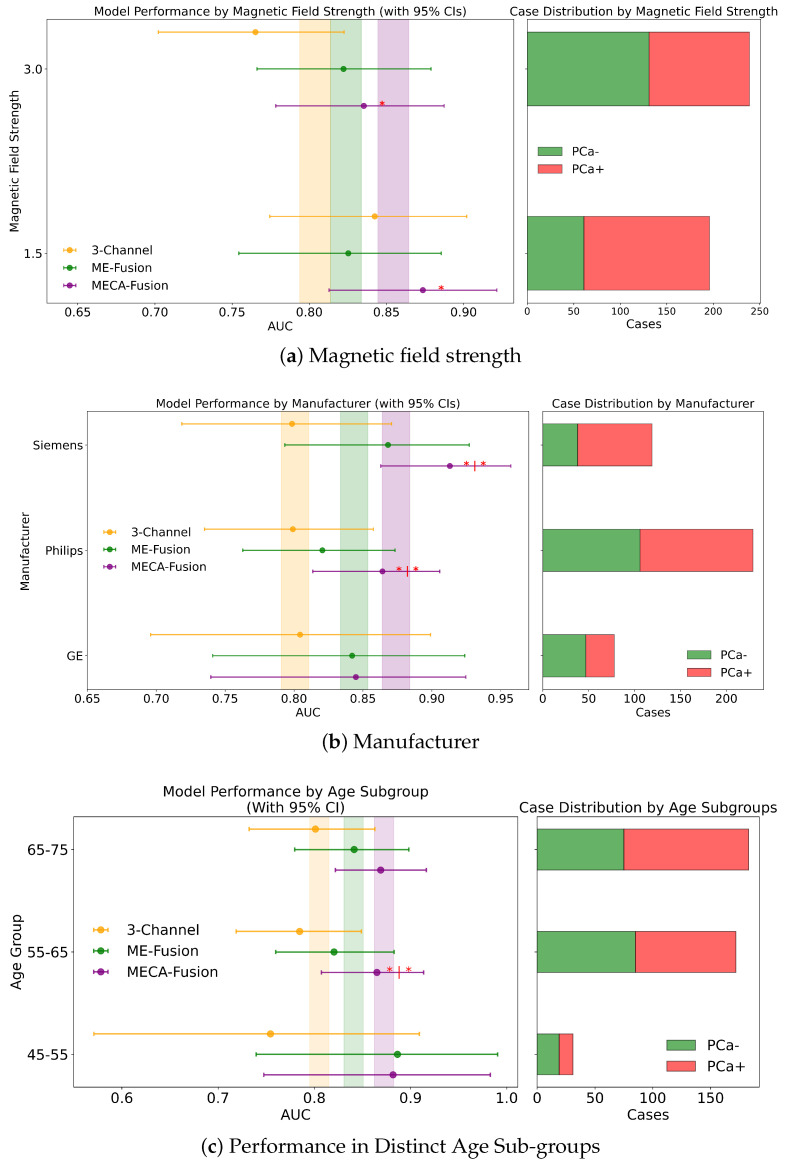
Fairness analysis for sub-cohorts stratified by (**a**) magnetic field strength, (**b**) manufacturer, and (**c**) distinct age groups. Point-and-range plots show model performance (mean and 95% CI) with vertical lines indicating expected performance on the test set. Asterisks (*) indicate statistical significance compared to other models at p≤0.05. If no ‘|’ symbol is shown, significance is relative to the 3-channel model; if ‘|’ is present, it applies to both compared models.

**Distinct Age Sub Groups ([45–55), [55–65], (65–75])**. We analysed model performance across three distinct age subgroups: [45–55), [55–65], and (65–75]. Within the retrospective cohort that included imaging and clinical data, the MECA-Fusion model consistently outperformed the 3-Channel model in all age groups, achieving significance with *p*-values of <0.05, <0.05, and <0.001, respectively. Notably, it also significantly surpassed the ME-Fusion model in the [55-65] age group with a *p*-value of <0.01. When evaluating the retrospective cohort using only imaging data, the pattern generally held; however, the MECA model’s performance notably declined in the [45–55) group, where it failed to reach statistical significance and exhibited a substantial 7% drop in AUC. In the prospective cohort incorporating both imaging and clinical data (Figure A1c), MECA demonstrated significant improvements specifically in the [55–65] age group compared to both comparator models, with a *p*-value of <0.05. Interestingly, when clinical data were excluded, MECA remained superior against the 3-Channel model across all age subgroups and was particularly significant in the youngest group, [45–55), with a *p*-value of <0.05. This suggests that the exclusion of clinical data may enhance MECA’s performance in certain demographic segments.

#### 3.4.3. Dataset Provider

In a detailed examination across various data providers, the performance of the MECA-Fusion model is compared to the two baseline models, as seen in Figure 7c. For the retrospective hold-out test set incorporating both imaging and clinical data, the MECA-Fusion model showed prominent performance. For data coming from the RMH center, it achieved statistical significance against the 3-Channel model with a *p*-value < 0.01 and was marginally significant compared to the ME-Fusion model with a *p*-value of 0.052. At other centers, such as RadboudUMC, NCI, and FChampalimaud, the model was statistically significant compared to the 3-Channel model with *p*-values < 0.05, demonstrating its effectiveness in these environments. However, in other centers, while the MECA-Fusion model showed higher AUC scores, it did not reach statistical significance. When only imaging data were considered, the MECA-Fusion model for the RMH center became significant against the ME-Fusion model with a *p*-value < 0.05 and maintained significant improvements for the RadboudUMC data with a *p*-value shifting from <0.05 to <0.0001. Interestingly, the inclusion of clinical data appeared to hinder the MECA-Fusion model compared to the ME-Fusion model at FChampalimaud, suggesting a possible interaction effect between model performance and clinical variables at this site. Centers like JCC, IDIBGI, and FPO are short of cases to conduct a valid statistical analysis.

**Figure A2:**
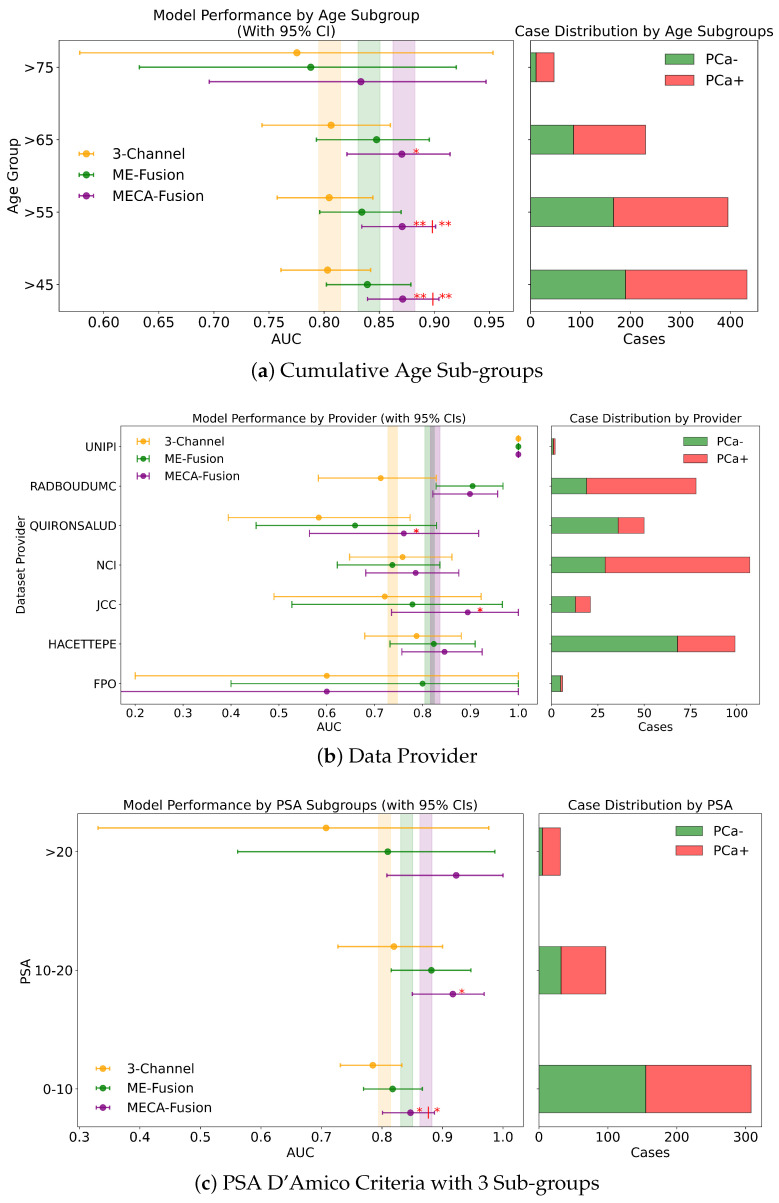
Fairnessanalysis for sub-cohorts stratified by (**a**) cumulative age groups, (**b**) data provider, and (**c**) PSA 4 sub-groups.Point-and-range plots show model performance (mean and 95% CI) with vertical lines indicating expected performance on the test set. Asterisks (*) indicate statistical significance, with ** (p≤0.01), * (p≤0.05). If no ‘|’ is shown, significance is relative to the 3-channel model; if ‘|’ is present, it applies to both compared models.

**Figure A3:**
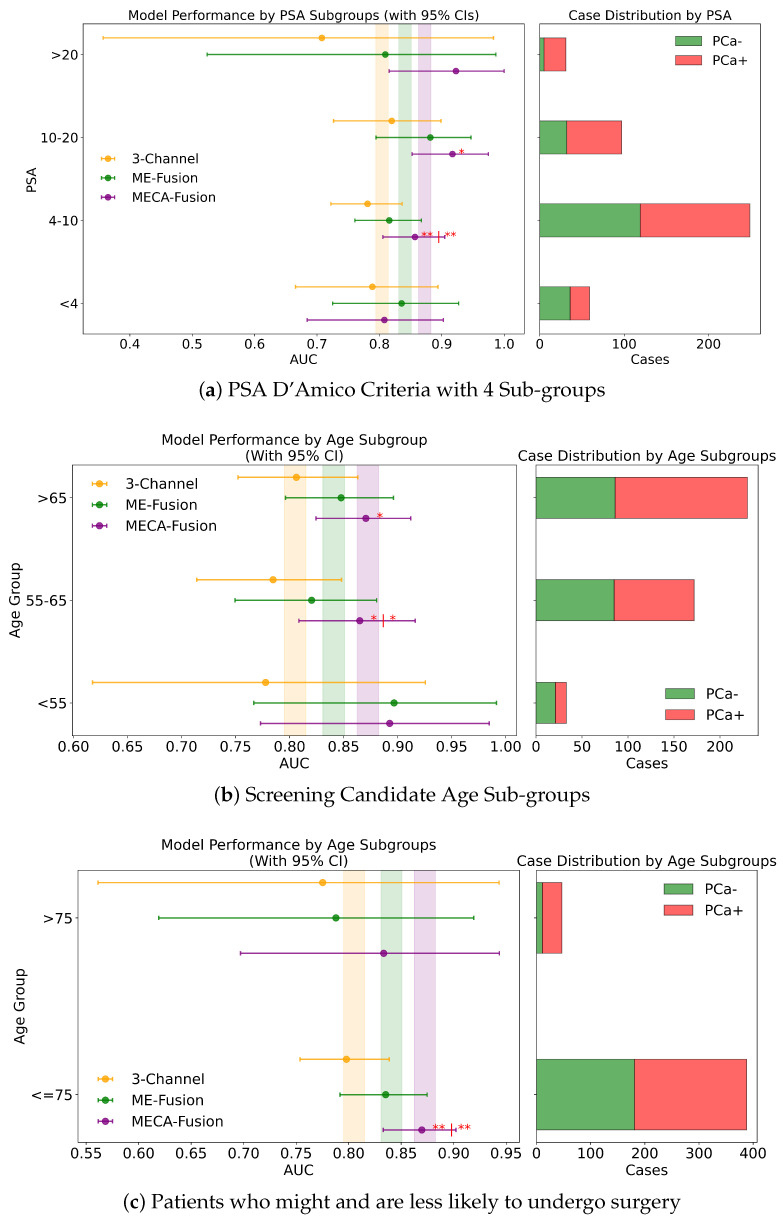
Fairness analysis for sub-cohorts stratified by (**a**) PSA 3 sub-groups, (**b**) screening candidate groups, and (**c**) surgical likelihood. Point-and-range plots show model performance (mean and 95% CI) with vertical lines indicating expected performance on the test set. Horizontal bar plots display target and stratum counts. Asterisks (*) indicate statistical significance, with ** (p≤0.01) and * (p≤0.05). If no ‘|’ symbol is shown, significance is relative to the 3-channel model; if a ‘|’ is present, it applies to both compared models.

For the prospective hold out test set, with both imaging and clinical data (Figure A2b), the MECA-Fusion model demonstrated statistical significance for the Quironsalud and JCC centers against the 3-Channel model with *p*-values < 0.05. However, when only imaging data are considered, the significance for the Quironsalud center was not maintained, but it continued to show significant improvement for the JCC center. Again, centers like FPO and UNIPI lacked sufficient data to conduct a valid statistical analysis.

#### 3.4.4. Magnetic Field

In an extensive evaluation focusing on bias and domain adaptation across different magnetic field strengths of MRI scanners, our MECA-Fusion model demonstrated varying levels of performance, dependent on both types of data (clinical and imaging versus solely imaging) and the retrospective or prospective cohorts.

For the retrospective cohort, when incorporating both imaging and clinical data, the MECA-Fusion model exhibited statistically significant improvements at both 1.5 tesla (*p* < 0.001) and 3 tesla (*p* < 0.001 compared to the 3-Channel model and *p* < 0.05 compared to the ME-Fusion model), as seen in Figure 7a. When only imaging data were considered, the performance of the MECA-Fusion model at 3 tesla remained significantly better than the 3-Channel model (*p* < 0.0001), although it did not show significant differences when compared to the ME-Fusion model. At 1.5 tesla with only imaging data, the MECA-Fusion model did not achieve statistical significance compared to the other models, yet it recorded a higher AUC.

For the prospective cohort analysis using both imaging and clinical data (Figure A1a), the performance of the MECA-Fusion model was significantly better compared to the 3-Channel model at both 1.5 and 3 tesla (*p* < 0.05). The performance was nearly significant when compared to the ME-Fusion model, with *p*-values closely approaching the threshold of significance (*p* = 0.054 and *p* = 0.056, respectively). However, when only imaging data were assessed, the MECA model showed statistical significance at 3 tesla against the 3-Channel model (*p* < 0.05) but did not demonstrate statistical significance at 1.5 tesla.

These findings underline that our proposed MECA-Fusion model has the capacity to adapt across different domains, particularly highlighting its robustness and superior performance in settings with varying magnetic field strengths.

#### 3.4.5. Manufacturer

Examining the performance across different MRI scanner manufacturers in the retrospective cohort using both imaging and clinical data, the MECA-Fusion model demonstrated significant improvements over the baseline models, 3-Channel and ME-Fusion, particularly on Siemens equipment, where it achieved *p*-values of less than 0.001 and 0.01 respectively. On Philips scanners, it also showcased significant superiority, achieving *p*-values of less than 0.01 against the 3-Channel and less than 0.05 against the ME-Fusion. Meanwhile, on GE equipment, the MECA-Fusion model was statistically significantly better than the 3-Channel model (*p* < 0.05) and approached significance against the ME-Fusion model (*p* = 0.055), as seen in Figure 7b. When imaging data only are considered, the MECA-Fsuion model maintained its significant performance on Siemens against both baseline models and exhibited higher AUC values on Philips and GE, though it did not reach statistical significance.

The prospective cohort analysis further confirmed these findings, with the MECA-Fusion model showing significant performance improvements on Siemens and Philips when integrating both imaging and clinical data (Figure A1b), with *p*-values less than 0.05. Interestingly, including clinical data enhanced the performance of the MECA-Fusion model, particularly on Siemens and Philips, where it remained statistically significant, (from *p*-value < 0.0001 to *p*-value < 0.05) when compared solely against the 3-Channel model. This observation suggests that the inclusion of clinical variables not only increases the efficacy of the MECA-Fusion model, but also moderately enhances the comparative performance against the baseline models.

## 4. Discussion

In the overall assessment of the model’s effectiveness across the entire ProstateNET dataset, the proposed Multi-Encoder-Cross-Attention-Fusion model demonstrated high performance across both retrospective and prospective test cohorts. In the retrospective cohort, MECA-Fusion with clinical data achieved the highest AUC (0.90), sensitivity (0.91), PPV of 83.75% and NPV of 83.46%, surpassing other models and radiologists’ assessments. Similarly, in the prospective cohort, MECA-Fusion with clinical data led with an AUC of 0.87, sensitivity (0.86), PPV of 79.04% and NPV of 83.02%. Statistical analysis using the DeLong tests confirmed the significant superiority of the MECA-Fusion model over the two baseline models for both cohorts. In the retrospective cohort, our proposed model showed significant improvements over both the ‘3-Channel’ and ‘Multi-Encoder-Fusion’ models, especially when clinical data were included. Notably, the inclusion of clinical data consistently enhanced the performance across all model variations, justifying the value of integrating clinical information. Similarly, in the prospective cohort, the MECA-Fusion model consistently outperformed both baseline models. These findings highlight the model’s robust capability to leverage both imaging and clinical data for improved diagnostic performance. These findings highlight our proposed model’s ability to generalize to out-of-distribution data with a significantly reduced need for expert annotated data from the new clinical setting.

### 4.1. Value of Clinical Data

We can see that clinical data largely enhance the performance of the DL models. However, for the MECA-Fusion model, the inclusion of clinical data does not consistently improve outcomes across all PSA subgroups. Specifically, in the highest PSA category (>20), the inclusion of clinical data does not result in statistical significance, suggesting that in cases with higher PSA levels, clinical data might not complement the model. Similarly, in the lowest PSA risk category (<4), an in-depth view in the prospective cohort without the use of clinical data shows that the model’s performance increases significantly, highlighting that clinical data can undermine performance for specific categories. A similar pattern is observed in the dataset-provider analysis. For the RMH data provider, the MECA-Fusion model performed better when using imaging data only. Including clinical data there seems to slightly degrade the performance relative to the ME-Fusion model.

### 4.2. Critical Cases

The MECA-Fusion model exhibits robust performance across different age groups, especially in cohorts where it tends to show statistically significant improvements over other models. In sub-cohorts divided by manufacturer and magnetic field strengths, the MECA-Fusion model generally performs better with clinical data, notably achieving significant results on Siemens and Philips machines, indicating its adaptability to varying vendors.

The performance of the MECA-Fusion model is notably higher in younger patients across different stratifications. The model achieves significant AUC values and statistical significance in these groups, indicating its utility in screening and early detection scenarios where younger patient populations are involved.

Out-of-distribution data are crucial for evaluating the generalisation ability of DL models. Our MECA-Fusion model demonstrates promising results, by retaining high AUC values and showing statistical significance in younger age categories and specific PSA groups when clinical data are included. This signifies that the MECA-Fusion model can adapt and perform well on out-of-distribution data, a prerequisite for deployment in real world situations.

### 4.3. Design Choices and the Role of the Cross-Modality Fusion Mechanism

In Section 2.3.3, we note that PCa typically appears as a hypointense region on T2-weighted images, a hyperintense region on high b-value DWI, and an area of low values on the corresponding ADC maps. These distinct appearances motivated the introduction of our proposed cross-attention mechanism to leverage the complementary features from each sequence.

Our initial approach employed a single encoder shared by T2w, DWI, and ADC inputs. Although this configuration already outperformed the 3-Channel baseline, it did not yield substantial gains for either the Multi-Encoder (ME) Fusion or the MECA-Fusion variants. To address this limitation, we incorporated three separate encoders—one for each sequence—so that each network branch could learn more sequence-specific representations. Coupled with the cross-attention mechanism, this multi-encoder strategy led to significantly improved performance, particularly in the ME-Fusion and MECA-Fusion models.

Notably, ME-Fusion alone often achieved results comparable to those of the MECA-Fusion model, indicating the effectiveness of specialised encoders in capturing the heterogeneity of PCa representation. Nonetheless, both ME-Fusion and MECA-Fusion consistently outperformed any single-encoder architecture, underscoring the value of combining sequence-specific feature extraction with cross-attention for robust diagnostic accuracy.

## 5. Conclusions

In this study, we introduced a novel multi-encoder-cross-attention 3D architecture for assessing PCa presence in whole bi-parametric MRI volumes. With an architecture specifically designed to exploit complementary features and the ProstateNET Imaging Archive, the largest image database worldwide of PCa mpMRI data, we established new baselines of performances. We evaluate our approach on a large separate prospective real-world cohort (436 exams), demonstrating that our method can perform better than standard clinical approaches and remains more robust to MRI bias field artifacts. Finally, a thorough analysis on fairness for different sub-cohorts was conducted.

## Figures and Tables

**Table 1:** Patient-level classification performance on the ProstateNET’s retrospective test cohort. bold highilights the best performing model.

Model	AUC (95% CI)	Sensitivity	Specificity	PPV	NPV
radiologists	0.82	0.83	0.71	81.95%	72.48%
3-Channel	0.82 (0.78–0.84)	0.85	0.62	78.01%	72.27%
3-Channel+clinical	0.85 (0.82–0.87)	0.89	0.62	78.79%	78.04%
ME-Fusion	0.85 (0.83–0.87)	0.81	**0.74**	83.17%	71.06%
ME-Fusion+clinical	0.87 (0.84–0.89)	0.86	0.69	81.48%	75.65%
MECA-Fusion	0.86 (0.84–0.89)	0.86	0.7	81.97%	75.92%
MECA-Fusion+clinical	**0.90 (0.88–0.91)**	**0.91**	0.72	**83.75%**	**83.46%**

**Table 2:** Patient-level classification performance on the ProstateNET’s prospective test cohort.

Model	AUC (95% CI)	Sensitivity	Specificity	PPV	NPV
radiologists	0.76	0.87	0.65	73.15%	82.02%
3-Channel	0.71 (0.66–0.75)	0.71	0.53	62.34%	62.51%
3-Channel+clinical	0.80 (0.76–0.84)	0.78	0.67	72.15%	73.54%
ME-Fusion	0.80 (0.75–0.84)	0.75	0.71	73.92%	72.15%
ME-Fusion+clinical	0.84 (0.80–0.87)	0.78	**0.77**	78.80%	76.15%
MECA-Fusion	0.83 (0.78–0.86)	0.80	0.73	76.46%	76.91%
MECA-Fusion+clinical	**0.87 (0.83–0.90)**	**0.86**	0.75	**79.04%**	**83.02%**

## Data Availability

Due to privacy and confidentiality agreements, we cannot currently share the dataset. However, we are exploring mechanisms to make the dataset available when public data sharing agreements will be finalised (2025).

## Appendix A. Code Availability

The relevant code and checkpoints are publicly available at: https://github.com/VinDimitriadis/PCa-classification (accessed on 18 March 2025)

## Appendix B. List of ProCAncer-I Consortium Members

**FORTH Institute of Computer Science-Computational BioMedicine Lab, Greece:** Stelios Sfakianakis, Varvara Kalokyri, Eleftherios Trivizakis
**FORTH-Institute of Molecular Biology and Biotechnology (FORTH-IMBB/BR), Greece:** Nikolaos Tachos, Eugenia Mylona, Dimitris Zaridis, Charalampos Kalantzopoulos
**Champalimaud Foundation, Portugal:** José Guilherme de Almeida, Ana Castro Verde, Ana Carolina Rodrigues, Nuno Rodrigues, Miguel Chambel
**Radboud, Netherlands:** Henkjan Huisman, Maarten de Rooij, Anindo Saha, Jasper J. Twilt, Jurgen Futterer
**HULAFE-Biomedical Imaging Research Group, Instituto de Investigación Sanitaria La Fe, Spain:** Luis Martí-Bonmatí, Leonor Cerdá-Alberich, Gloria Ribas, Silvia Navarro, Manuel Marfil
**University of Pisa, Italy:** Emanuele Neri, Giacomo Aringhieri, Lorenzo Tumminello, Vincenzo Mendola
**Hacettepe-Department of Radiology, Turkey:** Deniz Akata, Mustafa Özmen, Ali Devrim Karaosmanoglu, Firat Atak, Musturay Karcaaltincaba
**Institute of Biomedical Research of Girona Dr. Josep Trueta (IDIBGI), Spain:** Joan C. Vilanova
**National Cancer Institute, Vilnius, Lithuania:** Jurgita Usinskiene, Ruta Briediene, Audrius Untanas, Kristina Slidevska
**General Anti-Cancer and Oncological Hospital of Athens, Greece:** Katsaros Vasilis, Georgiou Georgios
**Radiology & AI Research Hub, The Royal Marsden NHS Foundation Trust, London, UK:** Dow-Mu Koh, Robby Emsley, Sharon Vit, Ana Ribeiro, Simon Doran, Tiaan Jacobs
**Quirónsalud Hospital/CIBERSAM, Valencia, Spain:** Gracián García-Martí
**Candiolo Cancer Institute, FPO-IRCCS, Turin, Italy:** Valentina Giannini, Giovanni Cappello, Giovanni Maimone, Valentina Napolitano
**Institute of Information Science and Technologies, National Research Council of Italy, Italy:** Sara Colantonio, Maria Antonietta Pascali, Eva Pachetti, Giulio del Corso, Danila Germanese, Andrea Berti, Gianluca Carloni
**Mass General Hospital, Boston, MA, USA:** Jayashree Kalpathy-Cramer, Christopher Bridge
**B3D, UK:** Joao Correia, Walter Hernandez
**Advantis, Greece:** Zoi Giavri, Christos Pollalis, Dimitrios Agraniotis
**Quibim, S.L., Valencia, Spain:** Ana Jiménez Pastor, Jose Munuera Mora
**Univie, Austria:** Clara Saillant, Theresa Henne, Rodessa Marquez

## References

[1] Sung H., Ferlay J., Siegel R.L., Laversanne M., Soerjomataram I., Jemal A., Bray F. (2021). Global cancer statistics 2020: GLOBOCAN estimates of incidence and mortality worldwide for 36 cancers in 185 countries. CA Cancer J. Clin..

[2] Siegel R.L., Miller K.D., Jemal A. (2018). Cancer statistics, 2018. CA Cancer J. Clin..

[3] Torre L.A., Bray F., Siegel R.L., Ferlay J., Lortet-Tieulent J., Jemal A. (2015). Global cancer statistics, 2012. CA Cancer J. Clin..

[4] Wang S., Liu X., Zhao J., Liu Y., Liu S., Liu Y., Zhao J. (2021). Computer auxiliary diagnosis technique of detecting cholangiocarcinoma based on medical imaging: A review. Comput. Methods Prog. Biomed..

[5] Sandhu G.S., Andriole G.L. (2012). Overdiagnosis of prostate cancer. J. Natl. Cancer Inst. Monogr..

[6] Hassanzadeh E., Glazer D.I., Dunne R.M., Fennessy F.M., Harisinghani M.G., Tempany C.M. (2017). Prostate imaging reporting and data system version 2 (PI-RADS v2): A pictorial review. Abdom. Radiol..

[7] Rosenkrantz A.B., Ginocchio L.A., Cornfeld D., Froemming A.T., Gupta R.T., Turkbey B., Westphalen A.C., Babb J.S., Margolis D.J. (2016). Interobserver reproducibility of the PI-RADS version 2 lexicon: A multicenter study of six experienced prostate radiologists. Radiology.

[8] Krizhevsky A., Sutskever I., Hinton G.E. (2012). Imagenet classification with deep convolutional neural networks. Adv. Neural Inf. Process. Syst..

[9] Long J., Shelhamer E., Darrell T. Fully convolutional networks for semantic segmentation. Proceedings of the IEEE Conference on Computer Vision and Pattern Recognition.

[10] He K., Zhang X., Ren S., Sun J. Deep residual learning for image recognition. Proceedings of the IEEE Conference on Computer Vision and Pattern Recognition.

[11] Twilt J.J., van Leeuwen K.G., Huisman H.J., Fütterer J.J., de Rooij M. (2021). Artificial intelligence based algorithms for prostate cancer classification and detection on magnetic resonance imaging: A narrative review. Diagnostics.

[12] Mehta P., Antonelli M., Ahmed H.U., Emberton M., Punwani S., Ourselin S. (2021). Computer-aided diagnosis of prostate cancer using multiparametric MRI and clinical features: A patient-level classification framework. Med. Image Anal..

[13] Wang X., Yang W., Weinreb J., Han J., Li Q., Kong X., Yan Y., Ke Z., Luo B., Liu T. (2017). Searching for prostate cancer by fully automated magnetic resonance imaging classification: Deep learning versus non-deep learning. Sci. Rep..

[14] Yoo S., Gujrathi I., Haider M.A., Khalvati F. (2019). Prostate cancer detection using deep convolutional neural networks. Sci. Rep..

[15] Aldoj N., Lukas S., Dewey M., Penzkofer T. (2020). Semi-automatic classification of prostate cancer on multi-parametric MR imaging using a multi-channel 3D convolutional neural network. Eur. Radiol..

[16] Armato III S.G., Huisman H., Drukker K., Hadjiiski L., Kirby J.S., Petrick N., Redmond G., Giger M.L., Cha K., Mamonov A. (2018). PROSTATEx Challenges for computerized classification of prostate lesions from multiparametric magnetic resonance images. J. Med. Imaging.

[17] Karagoz A., Alis D., Seker M.E., Zeybel G., Yergin M., Oksuz I., Karaarslan E. (2023). Anatomically guided self-adapting deep neural network for clinically significant prostate cancer detection on bi-parametric MRI: A multi-center study. Insights Imaging.

[18] Pachetti E., Colantonio S., Pascali M.A. (2022). On the effectiveness of 3D vision transformers for the prediction of prostate cancer aggressiveness. Proceedings of the International Conference on Image Analysis and Processing.

[19] Gavade A.B., Nerli R., Kanwal N., Gavade P.A., Pol S.S., Rizvi S.T.H. (2023). Automated diagnosis of prostate cancer using mpmri images: A deep learning approach for clinical decision support. Computers.

[20] Jue J.S., Barboza M.P., Prakash N.S., Venkatramani V., Sinha V.R., Pavan N., Nahar B., Kanabur P., Ahdoot M., Dong Y. (2017). Re-examining prostate-specific antigen (PSA) density: Defining the optimal PSA range and patients for using PSA density to predict prostate cancer using extended template biopsy. Urology.

[21] Kundu S.D., Roehl K.A., Yu X., Antenor J.A.V., Suarez B.K., Catalona W.J. (2007). Prostate specific antigen density correlates with features of prostate cancer aggressiveness. J. Urol..

[22] Isensee F., Jaeger P.F., Kohl S.A., Petersen J., Maier-Hein K.H. (2021). nnU-Net: A self-configuring method for deep learning-based biomedical image segmentation. Nat. Methods.

[23] Cardoso M.J., Li W., Brown R., Ma N., Kerfoot E., Wang Y., Murrey B., Myronenko A., Zhao C., Yang D. (2022). Monai: An open-source framework for deep learning in healthcare. arXiv.

[24] ProstateNET Imaging Archive. https://prostatenet.eu/.

[25] Fassia M.K., Balasubramanian A., Woo S., Vargas H.A., Hricak H., Konukoglu E., Becker A.S. (2024). Deep learning prostate MRI segmentation accuracy and robustness: A systematic review. Radiol. Artif. Intell..

[26] Simonyan K., Zisserman A. (2014). Very deep convolutional networks for large-scale image recognition. arXiv.

[27] Kingma D.P., Ba J. (2014). Adam: A method for stochastic optimization. arXiv.

[28] Akobeng A.K. (2007). Understanding diagnostic tests 2: Likelihood ratios, pre-and post-test probabilities and their use in clinical practice. Acta Paediatr..

[29] DeLong E.R., DeLong D.M., Clarke-Pearson D.L. (1988). Comparing the areas under two or more correlated receiver operating characteristic curves: A nonparametric approach. Biometrics.

